# Decellularized matrix for repairing intervertebral disc degeneration: Fabrication methods, applications and animal models

**DOI:** 10.1016/j.mtbio.2022.100523

**Published:** 2022-12-17

**Authors:** Hu Qian, Li He, Zhimin Ye, Zairong Wei, Jun Ao

**Affiliations:** a Department of Orthopaedic Surgery, Affiliated Hospital of Zunyi Medical University, Zunyi, China; b Department of Pathology, School of Basic Medical Sciences, Central South University, Changsha, China; c Department of Burns and Plastic Surgery, The Affiliated Hospital of Zunyi Medical College, Zunyi, China

**Keywords:** Intervertebral disc degeneration, Tissue engineering, Decellularized matrix, Regenerative medicine, (LBP), Low back pain, (IDD), intervertebral disc degeneration, (ECM), extracellular matrix, (IVD), intervertebral disc, (DCM), decellularized matrix, (NP), nucleus pulposus, (AF), annular fibers, (sGAG), sulfated glycosaminoglycan, (iAF), inner annular fibers, (oAF), outer annular fibers, (DWJM), Wharton's jelly matrix, (SIS), small intestinal submucosa, (DET), detergent-enzymatic treatment, (SDS), sodium dodecyl sulfate, (DAPI), 4,6-diamidino-2-phenylindole, (SD), sodium deoxycholate, (hDF), human dermal fibroblast, (3D), three-dimensional, (hADSCs), human adipose-derived stem cells, (APNP), acellular hydrogel descendent from porcine NP, (NPCS), NP-based cell delivery system, (TGF), transforming growth factor, (bFGF), basic fibroblast growth factor, (PEGDA/DAFM), polyethylene glycol diacrylate/decellularized AF matrix, (AFSC), AF stem cells, (DAF-G), decellularized AF hydrogel, (Exos), exosome, (EVs), extracellular vesicles

## Abstract

Intervertebral disc degeneration (IDD)-induced low back pain significantly influences the quality of life, placing a burden on public health systems worldwide. Currently available therapeutic strategies, such as conservative or operative treatment, cannot effectively restore intervertebral disc (IVD) function. Decellularized matrix (DCM) is a tissue-engineered biomaterial fabricated using physical, chemical, and enzymatic technologies to eliminate cells and antigens. By contrast, the extracellular matrix (ECM), including collagen and glycosaminoglycans, which are well retained, have been extensively studied in IVD regeneration. DCM inherits the native architecture and specific-differentiation induction ability of IVD and has demonstrated effectiveness in IVD regeneration in vitro and in vivo. Moreover, significant improvements have been achieved in the preparation process, mechanistic insights, and application of DCM for IDD repair. Herein, we comprehensively summarize and provide an overview of the roles and applications of DCM for IDD repair based on the existing evidence to shed a novel light on the clinical treatment of IDD.

## Highlights

• The fabricating procedures of DCM for IDD repair have progressed considerably.
• DCM materials could be applied as plain DCM, processed into hydrogel, and reactivated using bioactive substance.
• They show excellent *in-vitro* and *in-vivo* therapeutic efficiency for IDD repair.
• Ideal IDD animal models need to be developed to evaluate the efficiency of DCM.

## Graphical abstract

Schematic illustrating role and application of decellularized matrix in repairing intervertebral disc degeneration. UC: Umbilical cord; AF: Annulus fibrosus; NP: Nucleus pulposus; IVD: Intervertebral disc; SIS: Small intestinal submucosa; DET: Detergent-enzymatic treatment.

**Figure:**
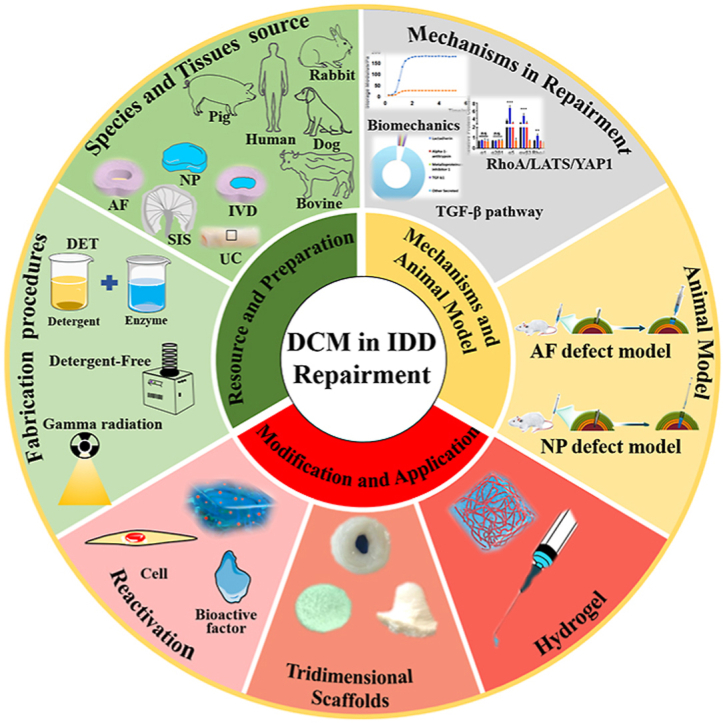

## 1 Introduction

Low back pain (LBP) affects many individuals, irrespective of sex, race, or socioeconomic status [1], causing a large economic burden to the global healthcare system. LBP has a high incidence, and epidemiological studies have shown that approximately 70–80% of people suffer from LBP, including neuropathic, nociceptive, non-specific, and nociplastic pain, at some point in their lives [[2], [3], [4]]. Generally, the most prevalent cause of LBP is intervertebral disc degeneration (IDD) caused by age, injury, genetics, and mechanical stress, resulting in lumbar disc herniation or other degenerative disc diseases [[5], [6], [7], [8]] and accounting for approximately 40% of LBP cases [9]. Current clinical treatments include conservative therapies, local blocking, and surgery. However, these methods do not effectively restore the physiological function [10,11].

Normally, the intervertebral disc (IVD) consists of a gelatinous core (nucleus pulposus [NP]) and a surrounding lamellar fibrocartilaginous ring (annulus fibrosus [AF]) [12]. Typically, no vascularity and nerves are present in the IVD, and the neighboring cartilaginous end plates supply its major nutrients through diffusion [11,13]. NP is abundant in sulfated glycosaminoglycans (sGAG), ranging from 300 to 600 ​μg/mg [[14], [15], [16]]. The IVD is a matrix-rich tissue with an extremely complex inner microenvironment and microstructure [17,18]. The extracellular matrix (ECM) of the IVD contains abundant collagenous and non-collagenous molecules possessing critical structural and regulatory functions.

As the most avascular tissue of the human body, the IVD is prone to degeneration [19]. IDD manifests as a reduction of the IVD height, decrease in the water content, thickening of the end plates, formation of fissures in annular fibers (AF), and ingrowth of nerve fibers [11,20,21]. IDD causes a debilitating injury to humans, and current management approaches are unsatisfactory [9,[22], [23], [24], [25], [26]]. Owing to the lack of self-healing capacity and extremely complex inherent architectures, designing an optimal regeneration strategy for IDD repair is challenging, especially when coupled with the complex process required to synthesize an artificial disc with the biomimetic mechanical and biological properties of the IVD [1]. The development of a novel therapeutic strategy for repairing IDD is urgently needed.

Recently, tissue engineering has drawn increasing attention [27,28]. Among various tissue-engineered scaffolds, decellularized matrix (DCM) is one of the most important materials [18]. DCM refers to biomaterials derived from human and animal organs or tissues with the removal of the immunogenic cellular components and maximal preservation of ECM via physical, chemical, and enzymatic technologies [[29], [30], [31]]. DCM has been applied in multiple organs and tissues, including the bladder, cornea, heart, liver, lung, skin, and tendon [1,[32], [33], [34]]. DCM possesses appropriate biomechanical properties, favorable regulatory molecules, and a microstructure mimicking the native IVD, thus functioning as a filling scaffold and delivery medium. Moreover, it was reported that cellular attachment, migration, and proliferation were closely associated with the ECM composition and structure, suggesting the potential differentiation induction role of DCM [35]. Compared with fully constructed grafts, DCM possesses a “prefabricated” microarchitecture approximating the native IVD without a complex additive-manufacturing operation, and the yielded scaffolds offer a “built-in” instructional induction to host cells, which causes them to produce tissue-specific phenotypes, contributing to IVD regeneration without adding exogenous bioactive factors [36].

Since the first report in 2011 [37], inspiring results have been reported in the field of DCM for IVD regeneration. Based on the evidence, we critically reviewed decellularized IVD repair scaffolds, including the optimization of decellularization methods, their mechanisms, applications, and animal models (**Graphical abstract**). This review further contributes to an understanding of DCM for IDD repair, providing an inspiring and novel strategy for the clinical management of IDD.

## 2 Optimization of decellularization procedures

### 2.1 Raw materials for DCM

**Fig. 1:**
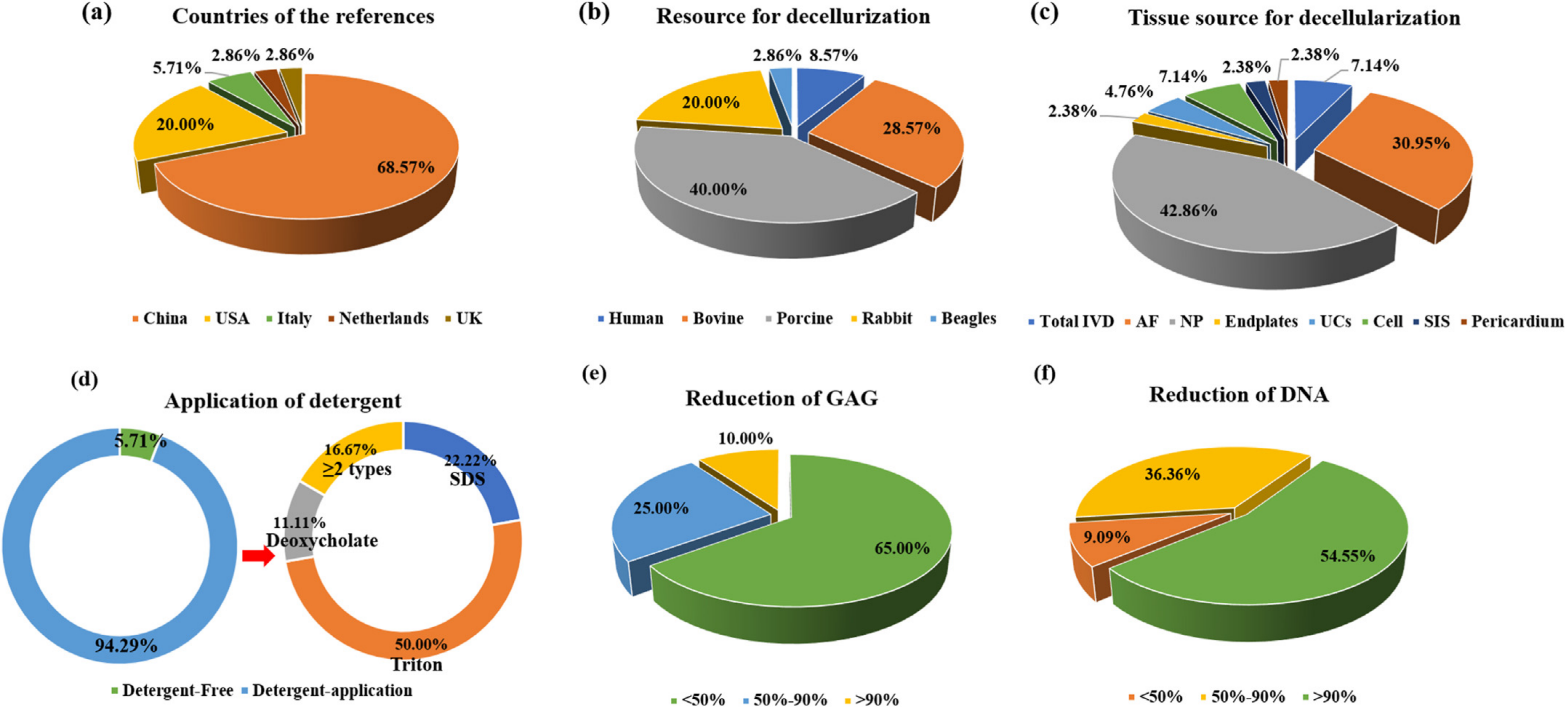
Diagram depicting the current status of decellularized matrix for repairing intervertebral disc degeneration. (a) The distribution of countries publishing relevant literatures. (b) The distribution of the races resource for decellularization. (c) The distribution of the tissue resource for decellularization. (d) The application of detergent used for decellularization. (e) Reduction of glycosaminoglycan during decellularization. (f) Reduction of DNA during decellularization.

Overall, in terms of the countries contributing to research on IDD repair, over half of the existing studies were reported by Chinese researchers (68.57%), followed by researchers from USA (20.00%) and Italy (5.71%) (Fig. 1a). All representative studies are listed in Table 1.

**Table 1:** Raw materials for decellularized materials for IVD repairing.

Author	Source	Decellularization Protocol (Treatment/Time)			
		Step 1	Step 2	Step 3	Step 4
Shan et al. [46]	Porcine SIS	Freeze-thaw cycle	2% Triton X-100/6 ​h	0.1% SDS/1 ​h	100 U/mL DNase/6 ​h
Wachs et al. [21]	Porcine CNP	SB-10/4 ​h	Triton X-200+SB-16/3 ​h	SB-10/1.75 ​h	75 U/ml Dnase+100μg/mL RNase/36 ​h
	Porcine TNP				
	Porcine LNP				
Lin et al. [38]	Rabbit AF	2% Triton X-100/24 ​h	1% SDS/24 ​h	200 U/mL DNase/12 ​h	PBS/6 ​h
	Rabbit NP				
Peng et al. [6]	Bovine AF	2% Triton X-100/NA	1% SDS/NA	200 U/mL DNase/NA	Freeze-drying
	Bovine NP				
Norbertczak et al. [39]	Bovine iAF	Freeze-thaw cycle	10 KIU/mL aprotinin/NA	0.1% SDS/14 days	Nuclease/3 ​h
	Bovine oAF				
	Bovine NP				
	Bovine endplate				
Yuan et al. [35]	rNPC ​+ ​Collagen	50 ​mM SB-10/30min	100 ​mM SA/NA	0.6 ​mM SB-16 ​+ ​0.14% Triton X-200/30 ​min	PBS/NA
Pei et al. [121]	Porcine SDSC/NPC	0.5% Triton X-100 ​+ ​20 ​mM AH/5min	NA	NA	NA
McGuire et al. [40]	Porcine pericardium	DDH_2_O/24 ​h	0.15% Triton X-100 ​+ ​0.25% DA+0.1% EDTA ​+ ​0.02% SA/6 days	70% ethanol/10min	720 U/mL DNase+ 720 U/mL RNase/24 ​h
Penolazzi et al. [43]	Human UCs	DDH_2_O-NA-24 ​h	4% DA/4 ​h	2000 kU DNase/1 ​h	Dehydration

NP: nucleus pulposus; AF: annular fibers; oAF: outer annular fibers; iAF: inner annular fibers; AFSC: AF stem cells; SIS: small intestinal submucosa; CNP: cervical nucleus pulposus; TNP: thoracic nucleus pulposus; LNP: lumbar nucleus pulposus; SDS: sodium dodecyl sulfate; NPC: nucleus pulposus cell; SDSC: synovium-derived stem cells; UCs: umbilical cords.

Suitable raw materials are the primary premise for preparing the ideal DCM since the decellularized materials take advantage of the native structure and microenvironment of primitive tissues. Currently, regarding species used in research, porcine tissue ranked first (40.00%), followed by bovine (28.57%) and rabbit (20.00%) tissues, with beagle tissue ranking the lowest (2.86%) (Fig. 1b). With respect to specific tissues, more than 40% of studies decellularized the nucleus pulposus (NP), and 30.95% of studies focused on the AF, with the total IVD accounting for 7.14% (Fig. 1c).

**Fig. 2:**
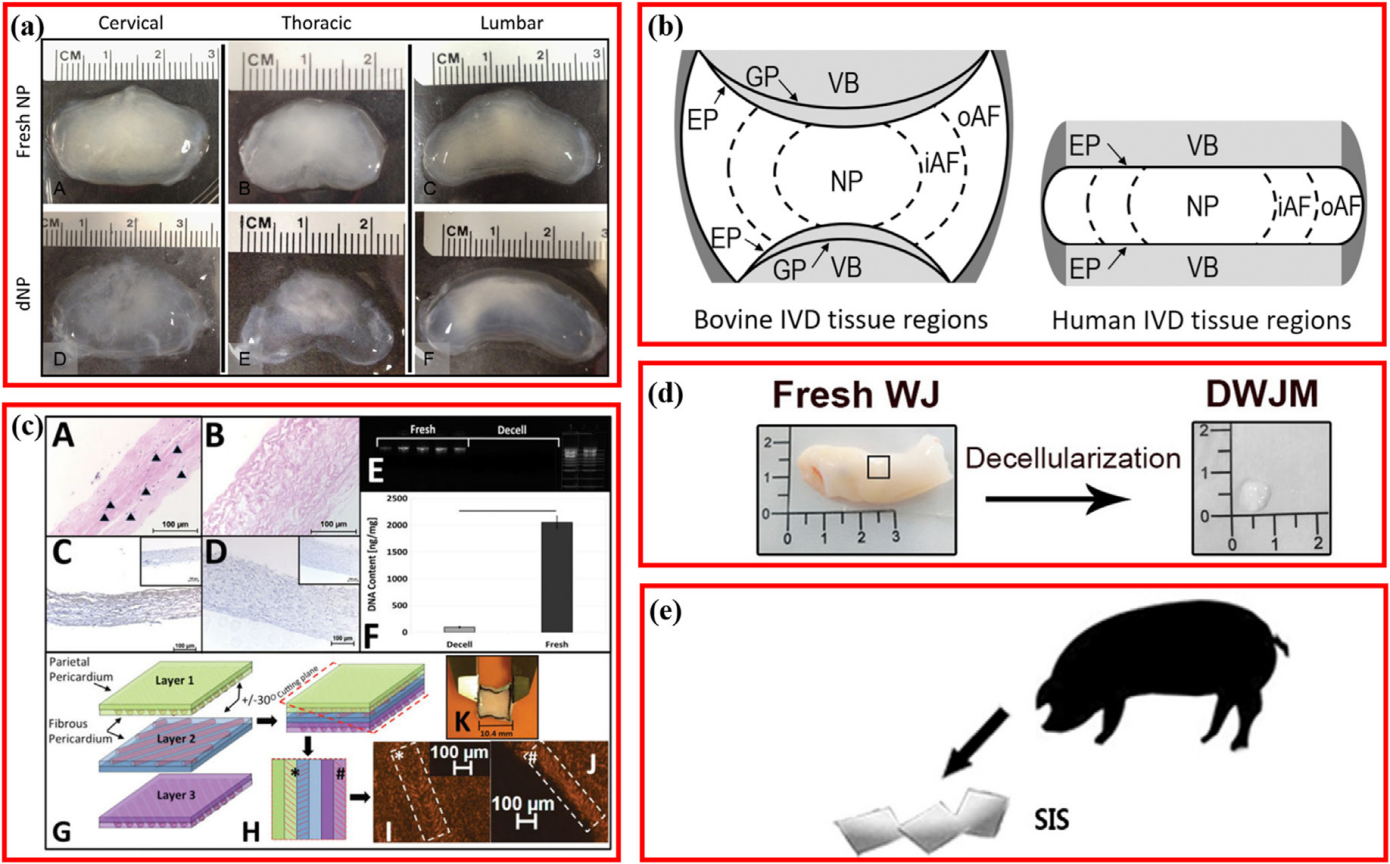
Raw materials for fabricating decellularized matrix repairing intervertebral disc (IVD) degeneration. (a) Nucleus pulposus derived from different segmental IVD. Adapted with permission from Ref. [21], copyright 2017 Elsevier Inc. (b) Schematic diagram depicting the IVD. Adapted with permission from Ref. [39], copyright 2020 Mary Ann Liebert Inc. (c) Fabrication and characterization of patch for annulus fibrosus repair using pericardial tissue. Adapted with permission from Ref. [40], copyright 2017 John Wiley and Sons Ltd. (d) Fabrication and characterization of patch for annulus fibrosus repair using pericardial tissue. Adapted with permission from Ref. [43], copyright 2020 Frontiers Media S.A. (e) Fabricating an injectable nucleus pulposus cell-modifed decellularized scaffold using small intestinal submucosa. Adapted with permission from Ref. [46], copyright 2017 Impact Journals LLC.

The selection and exploration of decellularized raw materials for IDD repair were carefully made. First, to clarify which spinal segment-derived IVD tissue was the most suitable for decellularization engineering, researchers extracted and decellularized NP of porcine cervical, thoracic, and lumbar IVD using the same method and compared the efficiencies (Fig. 2a) [21]. The results indicated a >96% DNA reduction in all segments, whereas sGAG maintenance was lowest in the thoracic segment, with no significant collagen content and cell removal differences between the cervical and lumbar NP. To explore the difference of decellularized NP and AF derived from different regions [21,38], further research comparing the inner annular fibers (iAF) and outer annular fibers (oAF) (Fig. 2b) revealed that the content of both total DNA and sGAG was higher in the iAF [39].

Some breakthroughs have also been made in the exploration of new materials. Native AF was characterized by angle-ply collagen architecture, with similar construction alignment to that found in the pericardium. Thus, the pericardium was explored to design multi-laminate angle-ply AF scaffolds (Fig. 2c) [40]. Owing to the merit of possessing a composition similar to the IVD, abundant hyaluronic acid, and growth factors [41,42], Wharton's jelly matrix (DWJM) derived from human umbilical cord was initially used for IDD repair through decellularizing (Fig. 2d) [43,44]. Likewise, since small intestinal submucosa (SIS) and NP share partial ECM with NP, enriched by the advantages of low cost and convenience to acquire, porcine SIS was chosen to fabricate the decellularized scaffold for IDD repair [45,46]. Thus, raw materials for decellularized IVD scaffolds were relatively mature, and several new materials are in the developmental stage.

Decellularization is a “top-down” process of fabricating biomaterial scaffolds, which is preceded by selecting appropriate raw materials. Presently, NP-derived DCM is the most reported (40%), and because NP is the most commonly affected part in IDD, researchers decellularize the NP or AF in the IVD to prepare the DCM with desirable architecture and specific-differentiation induction ability. Some other natural and synthetic forms were created to simulate the IVD microenvironment and architecture as IVD scaffolds [47,48], including simple scaffolds or gels [49,50] and complex AF, NP, and total IVD structures [[51], [52], [53]]. However, these materials showed limited use in IDD therapy since they possessed limited physical and mechanical properties intrinsic to the IVD owing to the lack of crucial functional factors replicating the IVD's native microenvironment [1]. For example, artificially synthesized AF could not confirm the NP region and could not sufficiently overcome the tensile loads [1], whereas NP scaffolds could not provide a gradual change in mechanical strength, similar to that by natural NP tissues [[54], [55], [56]]. Although DWJM [43], the pericardium [40], and SIS [46], being neoteric decellularization materials, were demonstrated to be promising agents for IDD repair, further validation is needed. NP- or AF-DCM could drive stem cells toward NP or AF cells, respectively [57]; however, both NP and AF cells are indispensable for proper IVD functioning. Moreover, researchers believe that single NP or AF-DCM may be insufficient for IVD regeneration; thus, we suggest the decellularization of the total IVD. It is noteworthy that some studies attempted to incorporate polymers such as chitosan and PECUU into the DCM to improve the bioactivity or mechanical property [58,59]. Chitosan possesses similar structures to sGAG, and its cationic properties help accumulate the high-valent anionic proteoglycan derived from cartilage or IVD cells, which favors the maintenance of the IVD function [60]. Adding PECUU improved the mechanical strength of the IVD [59]. Integration with polymers seems to be a promising approach for DCM modification for IDD repair. Thus, cells seeded in collagen or DCM were decellularized directly. Bioprinting provided novel access to make full use of DCM for repairing human tissue [[61], [62], [63]], and promising reports have been observed in IVD repair [64]. Additionally, multi-phasic decellularized composite scaffold comprising different tissues, exhibited a unique advantage in addressing the boundary between damaged tissue and adjacent tissues [65], which offered a well-developed inspiration for further research into IDD treatment.

### 2.2 Optimization of decellularization procedures

Fabrication technology is another element in determining the property and applicability of DCM. Presently, detergent-enzymatic treatment (DET) dominates DCM construction (94.29%) [43,44]. However, a detergent-free method has also been reported [21,66] (Fig. 1d). With respect to the detergents in various DETs, Triton (X-100 or X-200) is the most common (50.00%), followed by sodium dodecyl sulfate (SDS) (22.22%). The content of residual cells, DNA, sGAG, protein, and collagen were the essential indexes to measure the efficiency of decellularization. The cellular residue was usually quantified via 4,6-diamidino-2-phenylindole (DAPI) staining. DNA, protein, and sGAG contents were routinely detected using the DNeasy assay [67], BCA assay, and 1,9-dimethylmethylene blue assay, respectively [68]. Collagen was determined by quantifying the hydroxyproline content using the chloramine-T assay [69,70]. Recently, 65% of studies reported that the DCM preserved most sGAG with a reduction of <50%, although several studies reported that >90% of sGAG was removed during decellularization (Fig. 1e). Conversely, 54.55% of studies demonstrated that >90% of DNA was removed through decellularization. However, 9.09% of studies stated that the DNA reduction was below 50% (Fig. 1f).

**Fig. 3:**
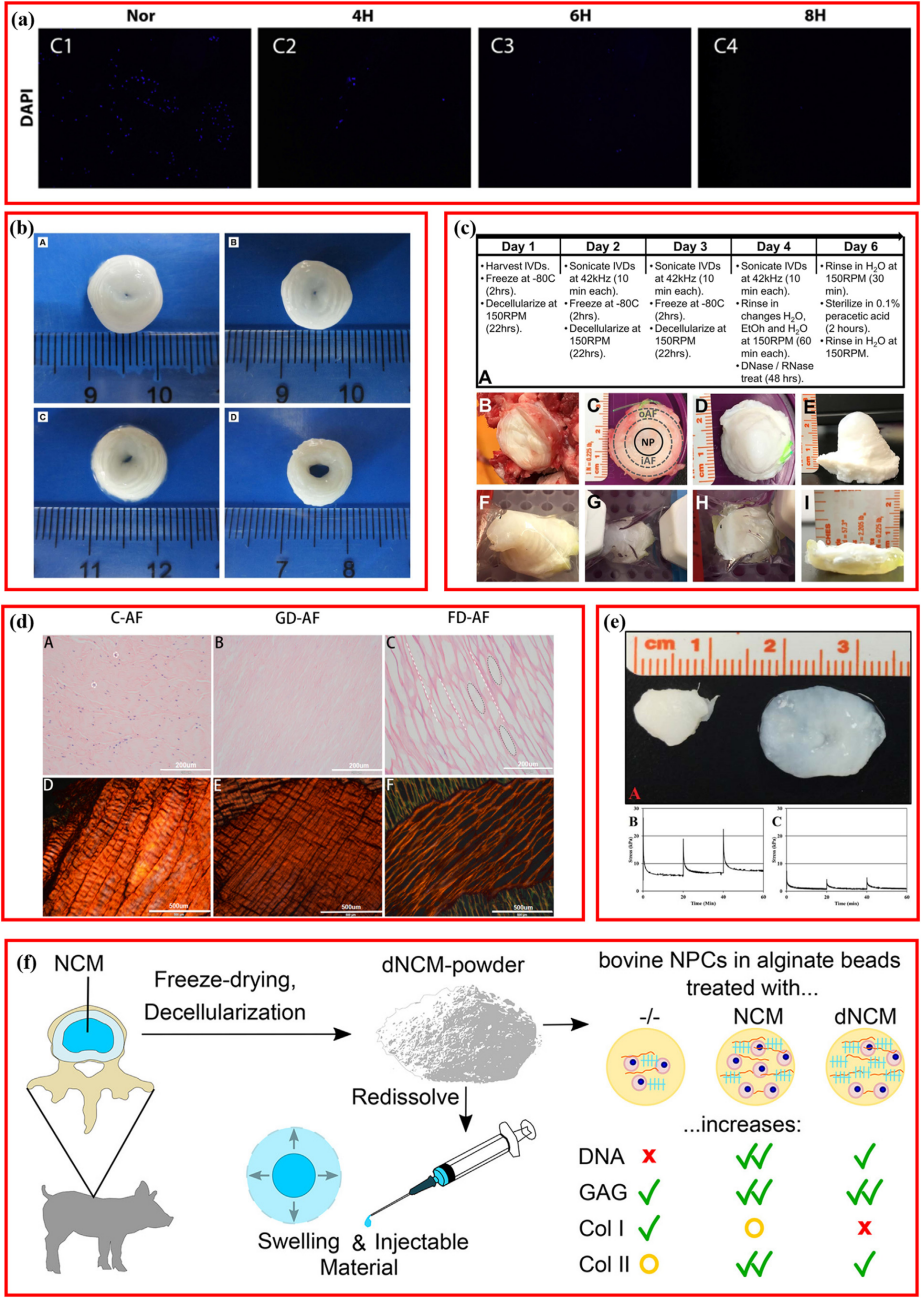
Optimization of decellularization methods. (a) DAPI staining of decellularised NP treated with SDS for different time. Adapted with permission from Ref. [71], copyright 2019 Elsevier. (b) Representative macroscopic images of annulus fibrosus before and after decellularization. Adapted with permission from Ref. [54], copyright 2014 Public Library of Science. (c) Schematic overview of whole bovine tail IVD decellularization process and time-line. Adapted with permission from Ref. [36], copyright 2018 John Wiley and Sons Inc. (d) Hematoxylin and eosin (H&E) staining and picrosirius red staining evaluating the decelluarization efficency. Adapted with permission from Ref. [73], copyright 2022 SAGE Publications Ltd. (e) Macroscopic image of nucleus pulposus and representative compression stress relaxation curves. Adapted with permission from Ref. [74], copyright 2016 John Wiley and Sons Inc. (f) Decellularized Notochordal cell-Derived matrix prepared using detergent-free method. Adapted with permission from Ref. [14], copyright 2022 American Chemical Society.

In the past decade, efforts were made to develop and optimize the procedures for preparing DCM for IDD repair, aiming at improving the above indexes (Table 2) [30]. Decellularization modification mainly focused on concentration, time, frequency, and intensity of reagent/treatment [1,35,37]. To optimize the detergent concentration used in decellularization, 0.05–0.6% Triton X-100 was applied to treat the porcine NP to indicate that complete cell removal could be achieved using increased concentration detergent with the incorporation of ultrasonication [37]. This thorough elimination was accomplished at the cost of sGAG reduction, although Triton X-100 concentration did not significantly affect the sGAG content when ultrasonication was used. However, it is worth noting that the phenomenon of the concentration of Triton X-100 not significantly affecting the sGAG content was only observed in low concentrations, ranging from 0.15% (v/v) to 0.6% (v/v) [37]. Furthermore, when the Triton X-100 concentration was increased, a 2% increment in concentration was suggested, which could preserve at least 80% of GAGs, while 3% Triton X-100 reduced the GAG content to approximately 65% [35]. For the same reason, 1% sodium deoxycholate (SD) was also recommended [35]. In addition, to assess the effect of working time on cellular removal, 0.5% SDS was used to treat the porcine NP for 4, 6, and 8 ​h, and the results suggested that fewer nuclei were observed in the longer treatment group (Fig. 3a) [71].

**Table 2:** Optimization of decellularization methods.

Author	Source	Decellularization Protocol (Treatment/Time)			
		Step 1	Step 2	Step 3	Step 4

Jin et al. [73]	Bovine AF	10 ​mM tris–HCl/2 ​h	Ethanol-70%/30min	0.2% Triton X-100 ​+ ​0.1% EDTA+10 KIU/mL aprotinin/24 ​h	50 U/mL DNase+1 U/mL RNase/3 ​h
Zhang et al. [89]	Rabbit NP	0.2%EDTA+0.3% Triton X-100 ​+ ​0.5% DA+1% PMSF/NA	70% ethanol/NA	750 mU/mL DNase+750 mU/mL RNase/30min	12,000 r/min centrifugation/5 ​min
Xu et al. [71]	Porcine NP	Freeze-thaw cycle	0.5% SDS/0 ​h	DNase/2 ​h	PBS/12 ​h
			0.5% SDS/4 ​h		
			0.5% SDS/6 ​h		
			0.5% SDS/8 ​h		
Hensley et al. [36]	Bovine IVD	1.2% Triton X-100 ​+ ​0.2% EDTA+ 0.02% SA/22 ​h	42 ​kHz ultrasonication/10 ​min	70% ethanol/30 ​min	720 mU/mL DNase+720 mU/mL RNase/48 ​h
Wu et al. [56]	Porcine AF	Freeze-thaw cycle	0.1% EDTA+10 KIU/mL aprotinin/24 ​h	0.1% SDS/24 ​h	50 U/mL DNase+1 U/mL RNase/3 ​h
Illien-Jünger et al. [72]	Bovine NP	2% SD	DNase	NA	NA
		2% SDS	2% SD	DNase	NA
		2% SDS	0.1% Triton X-100	2% SD	DNase
Huang et al. [81]	Human NP	Freeze-thaw cycle	2% SDS/24 ​h	0.25% trypsin+0.04% EDTA/0.5 ​h	3% Triton X-100/48 ​h
Fernandez et al. [74]	Bovine NP	DDH2O/24 ​h	1.2% Triton X-100 ​+ ​0.2% EDTA ​+ ​0.02% SA/3days	70% ethanol/10min	Nuclease/48 ​h
Xu et al. [54]	Porcine AF	0.1% EDTA+10 KIU/mL aprotinin/48 ​h	0.1%EDTA+10KIU/mL aprotinin+3%		
Triton X-100/72 ​h	0.2 μg/mL DNase+0.2 μg/mL RNase/24 ​h	PBS/24 ​h			
		Freezing-dissolving	0.1%EDTA+10 KIU/mL aprotinin+0.5% SDS/72 ​h	0.2 μg/mL DNase+0.2 μg/mL RNase/24 ​h	PBS/24 ​h
		0.5% trypsin+0.2% EDTA+0.2 ​mg/mL DNase+20 μg/mL RNase/24 ​h	PBS/24 ​h	NA	NA
Wu et al. [56].	Porcine AF	Freeze-thaw cycle	0.1% SDS+10 KIU/mL aprotinin/48 ​h	50 U/mL DNase+1 U/mL RNase/3 ​h	PBS/8 ​h
Ding et al. [66]	Beagles IVD	18-kGy Gamma irradiation	NA	NA	NA
		25-kGy Gamma irradiation			
		50-kGy Gamma irradiation			
Chan et al. [1]	Bovine IVD	0.1% SDS ​+ ​cocktail	Freeze-thaw cycle	PBS/24 ​h	NA
Schmitz et al. [14]	Porcine NP	200 U/mL benzonase/48 ​h	PBS/30min	Centrifugation-1000 ​g/5min	Freezing lyophilizing/72 ​h
Mercuri et al. [37]	Porcine NP	0.15% Triton X-100 ​+ ​0.25% DA/72 ​h	70% ethanol/10min	720 mU/mL DNase+720 mU/mL RNase/72 ​h	42 ​kHz ultrasonication/10 ​min
		0.10% Triton X-100 ​+ ​0.25% DA/72 ​h			NA
		0.05% Triton X-100 ​+ ​0.25% DA/72 ​h			NA
		0.6% Triton X-100 ​+ ​0.25% DA/72 ​h			42 ​kHz ultrasonication/10 ​min

NP: nucleus pulposus; AF: annular fibers; IVD: intervertebral disc; SDS: sodium dodecyl sulfate; DA: deoxycholic acid; EDTA: ethylenediamine tetraacetic acid; SD: Sodium deoxycholate.

A suitable detergent for decellularization was first explored [54,72]. Presently, the common detergents used for decellularization contain SDS, Triton X-100, and SD. In the same protocol, Triton X-100 was preferable for treating AF because it kept the major ECM components, tensile mechanical properties, and concentric lamellar structure and exhibited more favorable biocompatibility than SDS and trypsin (Fig. 3b) [54]. Furthermore, Illien-Jünger et al. explored the effect of adding one more detergent (SDS) or two more detergents (SDS ​+ ​Triton X-100) to base SD [72]. Contrasting the idea that more is better, they demonstrated that single SD performed best in depleting DNA, preserving collagen content and microstructure, and maintaining the GAG content. Moreover, decellularized NP, prepared using a single SD, showed the NP tissue composition and architecture closest to native ECM [72]. Some researchers modified their original procedures to decellularize the total IVD to match native human IVD in terms of mechanical characteristics, geometry, biochemistry, and microarchitecture via combing detergents, nucleases, freeze-thaw cycles, and ultrasonication, demonstrating the decellularized whole-IVD xenograft similar to native IVD, as an excellent substitute for IDD (Fig. 3c) [36]. Freeze-drying and gradient dehydration were common drying processes, but the method most beneficial for decellularizing AF remained unknown. Reportedly, freeze-drying preserved more hydrophilicity and aligned porous structures (Fig. 3d), showing a better ability to support hBMSCs growth than gradient dehydration [73]. In the most comprehensive modification research, 13 different procedures with varied treatment times of decellularization solution and ethanol, concentration of EDTA, Triton X-100, SDS, etc., were compared to optimize the decellularization technology [74]. The most effective program was identified as Triton X-100 (1.2%), sodium azide (0.02%), EDTA (0.2%), and bonding with ultrasonication (40 ​kHz) and nuclease treatment, which generated decellularized scaffold biomimetic with the natural NP tissue (Fig. 3e).

Except for optimization, the development of a new decellularization method was also performed. Gamma (γ) irradiation could damage cellular membranes directly and subsequently destroy the cellular structures, making decellularization controllable. Therefore, when applying different-intensity (18, 25, and 50 18-kGy) ^60^Co γ-irradiation to treat the IVDs of beagles, it is important to explore the feasibility of decellularizing IVD using γ irradiation and to evaluate the capacity for repairing IDD for the first time [66]. Results suggested that cell viability declined dramatically with the irradiated dose increasing, without significantly affecting biomechanical properties. However, in vivo results indicated that segmental degeneration in the high-dose irradiation group was more severe than that in the control and low-dose irradiation groups [66]. Moreover, because DET also has certain limitations and shortcomings, the detergent-free decellularization method was developed (Fig. 3f) [14], which involved treatment with sequential benzonase, centrifugation, and lyophilizing, and 93.9 ​± ​3.1% of DNA was removed efficiently; however, the loss in GAG and collagen content was not statistically significant.

As mentioned previously, the decellularization process is an important determining factor for the DCM. Therefore, the following DCM criteria have been proposed: (Ⅰ) the prepared DCM should permit DNA detection within <50 ​ng/mg and <200 bp length; (Ⅱ)no visible nuclear substances should be observed via H&E or DAPI staining [75,76]; and (Ⅲ) the ratio of sGAG-to-hydroxyproline in the NP tissue from healthy humans should be approximately 27:1 [77]. DCM was optimized to achieve these indexes. Different decellularization solutions comprising various reagents are used, which focus on breaking protein bonds, loosening the collagen network, allowing penetration of the solution into the intracellular layer, and denaturing the membrane-associated antigens [67,78,79].

Almost all studies (94.29%) used non-ionic detergents such as Triton X-100, and anionic detergents such as SDS to treat the materials. However, detergents change the structure and composition of decellularized tissues, which may inﬂuence the viability of the infiltrating cells and cause them to become cytotoxic [14,80]. Hence, it is important to develop a detergent-free decellularization procedure to considerably reduce the use of detergents. However, we must acknowledge that the newly-developed detergent-free decellularization processes showed certain drawbacks. Although γ rays can reduce cells effectively, poor therapeutic efficiency prevented IDD in vivo [66]. Furthermore, although another detergent-free method can significantly remove cells, sGAG and collagen preservation rates were not satisfactory [14].

Reagents with different concentrations ranges showed different effects on decellularization. For example, Triton X-100 with a concentration of <0.6% exhibited no effect on GAGs removal; however, it exhibited adverse effects at a concentration above 2% [35]. Combining multiple eluents did not produce better results [72]. The primary purpose of decellularizing tissues for preparing scaffolds is to remove all cells, antigenic epitopes, and nuclear substances while retaining critical ECM constituents in the inherent relative ratios, spatial distribution, and tissue microarchitecture, aiming to cut down the probability of immune rejection and fabricating a biomimetic scaffold [36,81,82]. Among the DCM, sGAG showed potent inhibition on neural ingrowth [83,84], whereas collagens were vital structural components of IVD; both were known to be essential for cellular adhesion and behavior [85]. All the procedures should be optimized to retain ECM and deplete cells and antigens; in decellularization, it is important to strike a balance between removing host cells and maintaining the inherent matrix [86]. During decellularization, the freeze-thaw cycle was employed in some studies for devitalizing cells, which may be attributed to the cellular lysis and fragmentation caused by the intracellularly formed ice crystals in rapid thawing and freezing [1]. Almost all studies used tissue-grinding to fragment the IVD for better decellularization of the tissue; however, whether this procedure would destroy the natural microstructure is unclear. The feasibility of loosening the architecture of ECM using ultrasonication has been demonstrated, revealing its applicability towards promoting the access of materials in and out of the matrix [87]. Although this technology is being modified gradually, the decellularization of total IVD remains a great challenge. To decellularize the total IVD, the end plate was thinned to within 2 ​mm with X-ray radiograph verification, and continuous irrigation was applied to remove the blood clots and debris for a longer duration due to the larger size and thickness [1] because vertebral bodies and clotted bony end plates may have been present [88].

All the parameters in the decellularization process were vital for efficiency, including the formula and concentration of the decellularized solution, ribozyme concentration, elution temperature, and time. During the optimization of DCM fabrication methods, the parameters were explored to search for the best value. The concentration of Triton X-100 should be no more than 2% [35,72], and 1% of Na deoxycholate concentration was suggested to preserve the GAG content [35]. A combination of multiple detergents was not recommended [72]. A freeze-thaw cycle and ultrasonication (42 kHz/10min) was suggested to be applied during the preparation of DCM [1,36,37,87]. The SDS treatment should be prolonged because a longer duration of SDS treatment was associated with a higher cellular removal rate, which was proved to be at least within 8 ​h [71]. With respect to the decellularization of IVD using γ irradiation, lower irradiation intensity was suggested to be used to prevent segmental degeneration [66]. Regarding the nuclease, the concentrations of DNase and RNase were optimized to 720 mU/mL-50 U/mL and 720 mU/mL-1 U/mL, both of which should be treated for 0.5–48 ​h [36,37,54,56,73,89]. In a limited range, extending the treatment time and increasing the reagent concentration can improve the cellular removal rate. However, caution should be exercised because these reagents may weaken the biomechanical property, which is important for IDD progression and repair. Moreover, meeting mechanical and biological compatibilities is necessary for the repair's efficacy and longevity [90]. Further studies should optimize the decellularization methods in a wider variable range, unifying the parameters and processes.

## 3 Application

Regenerative medicine for IDD repair has developed tremendously in the past decades, with DCM representing one of the most famous approaches involving both in vitro and in vivo experiments. A DCM has been applied for IDD repair in the form of multiple unique implants, including plain and processed DCM, the latter of which contained hydrogel-form DCM and composite DCM scaffolds reactivated using cell/bioactive factor.

### 3.1 Plain DCM

IDD repair with the plain DCM directly utilizes the natural macro-/micro-environment of IVD without disturbing the intricate complex structure. In addition, there is no concern about the additive active substance, and it is easy to prepare.

**Fig. 4:**
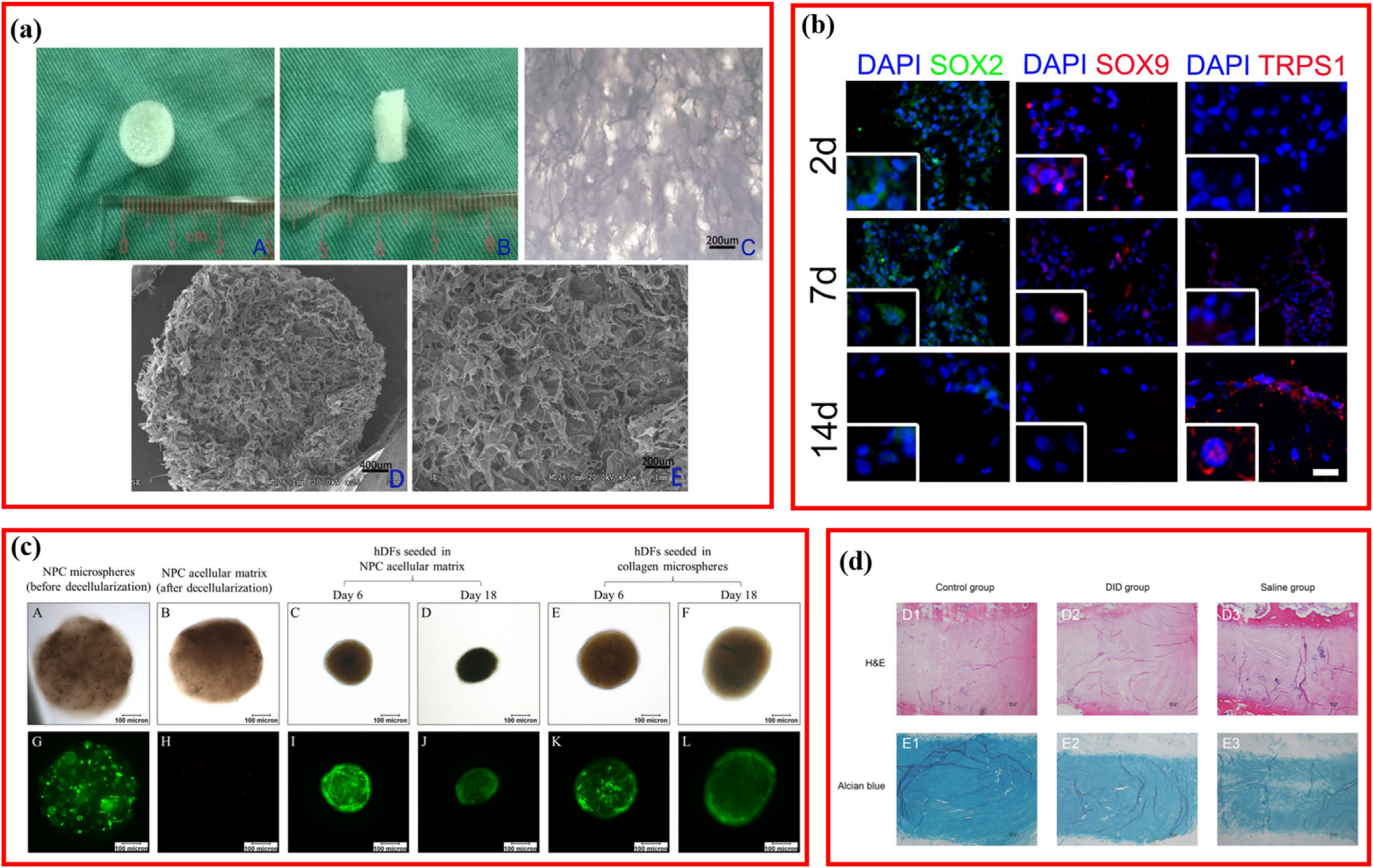
Plain decellularized matrix for repairing intervertebral disc degeneration (IDD). (a) Biomimetic scaffold derived from rabbit nucleus pulposus tissue. Adapted with permission from Ref. [89], copyright 2021 Springer US. (b) Representative macroscopic images of annulus fibrosus before and after decellularization. Adapted with permission from Ref. [43], copyright 2020 Frontiers Media S.A. (c) Viability of the NPC microspheres before and after decellularization, and of hDFs seeded in the NPC acellular matrix and collagen microspheres. Adapted with permission from Ref. [91], copyright 2018 Springer Nature. (d) H&E and Alcian blue staining of intervertebral disc at 2 months after injection. Adapted with permission from Ref. [38], copyright 2016 Impact Journals LLC.

Usually, DCM is used directly as tridimensional plain scaffolds (Table 3). Directly-used decellularized scaffolds were mainly derived from NP [37,72,89] and total IVD [1,38,66]. In vitro results suggested that decellularized scaffolds were biocompatible when seeded with rBMSCs, human dermal fibroblast (hDF), MSCs (WJ), and human ADSC [1,37,38,43,72,89,91]. The in vivo evaluation also revealed the feasibility and efficiency of DCM for IDD repair [38,66]. An interconnected three-dimensional (3D) porous scaffold derived from rabbit NP was prepared via sequential detergent-nuclease treatment, the porosity of which was as high as 81.28 ​± ​4.10% (Fig. 4a) [89]. Interestingly, after seeding rBMSCs, the compressive modulus increased as the culturing time prolonging, varying from 0.04 ​MPa (control) to 0.07 ​MPa (2 weeks) and 0.12 ​MPa (4 weeks), suggesting the ingrowth of rBMSCs and the integration between cells and decellularized scaffolds. Moreover, decellularized scaffolds produced from human umbilical cords were capable of inducing massive 3D cellular aggregation when cultured with DWJM-derived hMSC and IVD cells and improving the degenerative phenotype of human IVD cells by influencing the expression of critical molecules (SOX2, TRPS1, and SOX9) that positively regulate IVD homeostasis (Fig. 4b) [43]. Similarly, decellularized scaffolds derived from nucleus pulposus cells (NPCs) cultured in the collagen microsphere were well repopulated by hDF, and their cellular phenotypes were substituted with CA12 and collagen type II expression, which were upregulated significantly (Fig. 4c) [91]. Decellularized rabbit IVD was demonstrated to be effective in the treatment of the rabbit IDD model without evoking any obvious inﬂammatory reaction (Fig. 4d) [38].

**Table 3:** Plain DCM for repairing IVD.

Author	Year	Source	Compressive modulus	Cell	Animal Model	Findings
Zhang et al. [89]	201	Rabbit NP	0.04 ​MPa	rBMSCs	NA	3D porous scaffold derived from NP ECM is a potential biomaterial for the regeneration of NP tissues.
Penolazzi et al. [43]	2020	Human CDs	NA	MSCs (WJ)/IVD cells	NA	DWJM cpuld improve the degenerated phenotype of human IVD.
Yuan et al. [91]	2018	rNPC ​+ ​collagen	NA	Dermal fibroblast	NA	Fabricating a biomimetic ECM microenvironment of native NP tissue via collagen microencapsulation and decellularization.
Lin et al. [38]	2016	Rabbit IVD	18.41 ​kPa	HDCs/rMSCs	Rabbit/IVD degeneration	Decellularized IVD could induce MSCs to diﬀerentiate into IVD-like cells
Illien-Jünger et al. [72]	2016	Bovine NP	NA	Human NPC/MSC	NA	Decellularized ECM promoted adaptation of NP cells and MSCs
Ding et al. [66]	2014	Beagles IVD	67.06 ​kPa	NA	Beagle/IVD degeneration	Decellularize disc prepared by Gamma Irradiation was excellent materials for degenerative disc disease.
Chan et al. [1]	2013	Bovine IVD	NA	Bovine NPC	NA	Decellularized IVD has good potential for IVD bioengineering.
Mercuri et al. [37]	2011	Porcine NP	15.9 ​kPa,	human ADSC	NA	Decellularization of porcine NP exhibited potential for TE the human NP.

NP: nucleus pulposus; NPC: nucleus pulposus cell; IVD: intervertebral disc; ADSC: adipose-derived stem cell; WJ: Wharton's jelly.

Based on the above reports, direct utilization of plain DCM is beneficial for cellular adhesion, proliferation, and in vivo IDD repair.

### 3.2 Processed DCM

#### 3.2.1 Hydrogels-form DCM

Despite the advantages of DCM, the inability of plasticity restricted its wider application, and hydrogel-form matrices are potentially viable materials for IDD treatment. Hydrogel-form DCM possesses notable advantages. The injectable material could fill the irregularly shaped structure defects and supplement biological components, except for delivering bioactive elements using a fine-gauge needle via minimally invasive operation, making the therapy easier; in situ gelling hydrogel also provides load bearing for the NP [21]. Hence, an increasing number of scholars attempted to design injectable DCM hydrogel for IDD repair, as listed in Table 4 [3,11,14,20,21,92].

**Table 4:** Hydrogel-form DCM for repairing IVD.

Author	Year	Source	Injectable	In-site gelling	Cell	Swelling ratio	Findings
Schmitz et al. [14]	2022	Porcine NP	Yes	No	Bovine NPC	350%	dNCM could be used as the basis of disc regeneration.
Piening et al. [11]	2022	Porcine NP	Yes	No	NA	NA	Decellularized NP could prevent nerve growth into the native disc and NP in vivo.
Yu et al. [3]	2020	Bovine NP	Yes	No	ADSC	NA	Injectable hydrogel had low toxicity and inducible differentiation.
Peng et al. [20]	2020	Bovine AF	Yes	No	hBMSCs	NA	Genipin-crosslinked decellularized annulus fibrosus hydrogels exhibited good biocompatibility, bioactivity, and better mechanical strength than DAF-G.
Mercuri et al. [92]	2013	Porcine NP	Yes	No	hADSCs	NA	APNP hydrogels may serve as a suitable scaffold for NP tissue regeneration.
Wachs et al. [21]	2017	Porcine CNP	Yes	Yes	Human NPC	NA	This injectable tissue-specific matrix hydrogel was biocompatible for NPCs.
		Porcine TNP					
		Porcine LNP					

NP: nucleus pulposus; AF: annular fibers; NPC: nucleus pulposus cell; hADSC: human adipose-derived stem cell; CNP: cervical nucleus pulposus; TNP: thoracic nucleus pulposus; LNP: lumbar nucleus pulposus; hBMSCs: human mesenchymal stem cells; APNP: acellular porcine nucleus pulposus.

**Fig. 5:**
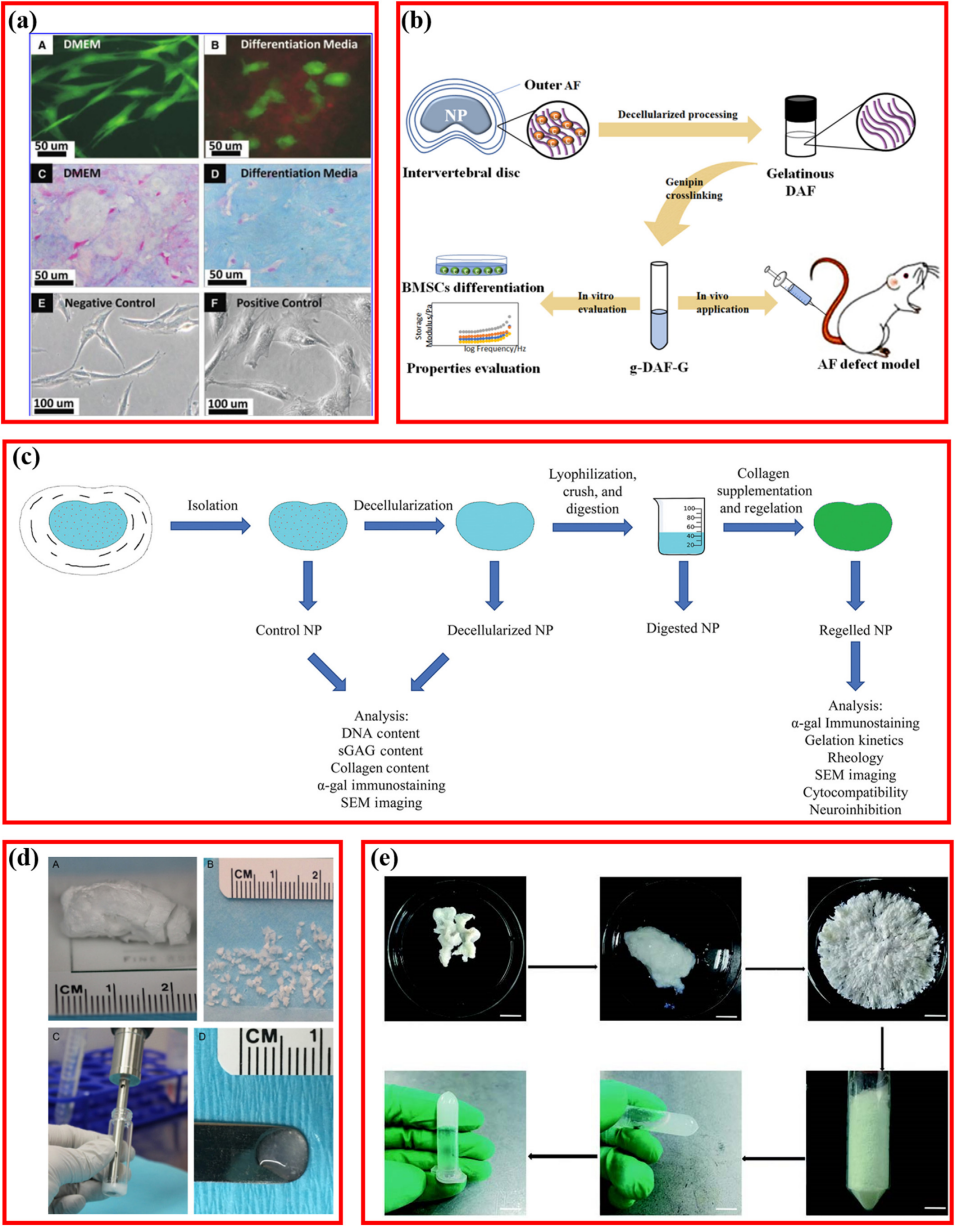
Hydrogel-form decellularized matrix for repairing intervertebral disc degeneration (IDD). (a) Representative LIVE/DEAD, Alcian blue staining and phase-contrast images of hADSCs cultured on decellularized porcine nucleus pulposus hydrogel. Adapted with permission from Ref. [92], copyright 2013 Mary Ann Liebert Inc. (b) Schematic illustrating the fabrication of genipin-crosslinked decellularized annulus fibrosus hydrogels. Adapted with permission from Ref. [20], copyright 2020 John Wiley and Sons Ltd. (c) Schematic illustration of the porcine nucleus pulposus decellularization. Adapted with permission from Ref. [11], copyright 2022 Wiley. (d) Pictorial illustration of the digest and re-gelation process of the decellularized nucleus pulposus. Adapted with permission from Ref. [21], copyright 2017 Elsevier Inc. (e) Schematic illustration of the fabrication of decellularized nucleus pulposus hydrogel and fresh nucleus pulposus. Adapted with permission from Ref. [3], copyright 2020 SAGE Publications Ltd.

Acellular matrix hydrogels extracted from porcine notochordal cells generate a swelling ratio as high as 350%, showing great potential as an injectable carrier of NPCs [14]. DCM hydrogels induced stem cell differentiation in AF or NPCs [3,20,92]. NP cell-positive gene markers were detected when human adipose-derived stem cells (hADSCs) were seeded on the acellular hydrogel produced from porcine NP (APNP) (Fig. 5a) [92]. Furthermore, the mechanical values of the hADSC-seeding hydrogel increased with time progressively, which was consistent with the data for natural NP tissue, suggesting that APNP hydrogels can be combined with autologous ADSCs as biomaterials for NP regeneration [92]. Similarly, hBMSCs expressed significantly higher levels of AF-specific genes, including *COL5A1, COL1A1, IBSP, TNMD,* and *FBLN1,* when sown onto AF-derived hydrogels (Fig. 5b) [20]. Furthermore, to improve the relatively low mechanical strength, the addition of optimized 0.02% genipin increased the storage modulus by approximately 7000-fold (465.5 ​Pa vs. 3.29 ​MPa) and restored the water content of NP in vivo [20]. sGAGs are potent neuroinhibitors, the loss of cause the initiation of IDD-derived pain [83,84]. Although it was extremely difficult to increase the sGAG content, another study demonstrated that an NP-derived acellular hydrogel formed at 37 ​°C markedly inhibited neuronal growth, similar to sGAG (Fig. 5c) [11]. As previously mentioned, injectable hydrogels could fill irregularly-shaped structural defects without implanting large scaffolds in open conditions. Hence, injectable hydrogels with in-site gelling capacity for NP repair were developed [3,21]. Wachs et al. reported the development of an acellular hydrogel gelling within 45 ​min in situ, which was able to form fibrillar collagen similar to native NP and promote sGAG formation without producing cytotoxic effects (Fig. 5d) [21]. Furthermore, Yu et al. demonstrated that an NP-derived acellular hydrogel could gel as a grid structure in a shorter duration (30 ​min) and that it increased the expression of NP-related genes significantly in addition to excellent in vitro and in vivo biocompatibility (Fig. 5e) [3].

These reports indicated that the development of DCM hydrogels opened a new window for IDD treatment.

#### 3.2.2 Composite DCM scaffolds reactivated using cell/bioactive factors

The inherent microenvironment of IVD is complex; it contains multiple types of cells and molecules. The “tissue engineering” comprises three critical elements, scaffolds, cells, and bioactive factors [[93], [94], [95]], an ideal regenerative IVD should include all three aspects to restore the function adequately. However, neither cells nor growth factors can survive after IDD initiation and progression [3,21]. Conversely, cells are almost fully eliminated during decellularization, alongside the inactivation of bioactive factors. Therefore, although the acellular matrix for IDD repair was partially bioactive, it lacked indigenous vigorous cells and high-efficiency bioactive factors, compared with native IVD tissues. Therefore, tissue engineering approaches employing bioactive factors and/or cells to equip the DCM for IDD repair seems advantageous (Table 5).

**Table 5:** DCM reactivated by cells or bioactive factors.

Author	Composite scaffold	Source	Hybrid Materials	Functional cell	Functional factor	Seeding Cell	Animal model	Findings
Liu et al. [58]	DAFM/chitosan hybrid hydrogels with bFGF	Rabbit AF	Chitosan	NA	bFGF	AFSCs	NA	Incorporation of bFGF into hydrogels promoted AF-related tissue synthesis.
Shan et al. [46]	DD-SIS	Porcine SIS	NA	Rabbit NPC	NA	NA	Rabbit/IVD degeneration	DD-SIS decellularized scaffold was safe and effective for treating IVD degeneration
Wei et al. [99]	PEGDA/DAFM/TGF-β1 hydrogels	Porcine AF	PEGDA	NA	TGF-β1	AF cells.	Rat/AF defect	TGF-β1-supplemented DAFM hydrogels hold promise for AF repair.
Yu et al. [96]	GDHA hydrogel	Bovine NP	Genipin	ADSCs	NA	NA	Rat/Disc degeneration	GDHA improved the cellular survival, intervertebral height, MRI index, and histological grading score
Liu et al. [112]	DAFM/PECUU	Rabbit AF	PECUU	NA	NA	AFSCs	Rat/Annulus fibrosus defects	DAFM/PECUU-blended scaffolds closely mimic the biological and mechanical properties of native tissue.
Yuan et al. [35]	rMSC-acellular NPC derived matrix	Rabbit NPC ​+ ​Col Ⅰ	NA	rMSCs	NA	NA	Rabbit/IVD degeneration	The composite scaffold could improve the GAGs and type II collagen.
Kuang et al. [60]	DNPM/chitosan	Rabbit NP	Chitosan	NA	TGF-β3	NPSCs	NA	DNPM/chitosan hybrid hydrogel was a potential scaffold for IVD degeneration.
Zhou et al. [15]	NPCS	Porcine NP	Chondroitin sulfate	hADSCs	NA	NA	Rabbit/IVD degeneration	The NPCS had good biological and mechanical properties and could promote the regeneration of degenerated NP.

PEGDA/DAFM: polyethylene glycol diacrylate/decellularized AF matrix; hADSCs: human adipose-derived stem cells; bFGF: basic fibroblast growth factor; TGF-β1: transforming growth factor β1; NPCS: NP-based cell delivery system; GDHA: genipin cross-linked decellularized nucleus pulposus hydrogel (GDH)-loaded adipose-derived mesenchymal stem cells; DNPM: decellularized nucleus pulposus matrix; DAFM/PECUU: decellularized annulus fibrosus matrix/poly(ether carbonate urethane)urea; DD-SIS: decellularized decellularized-small intestinal submucosa.

**Fig. 6:**
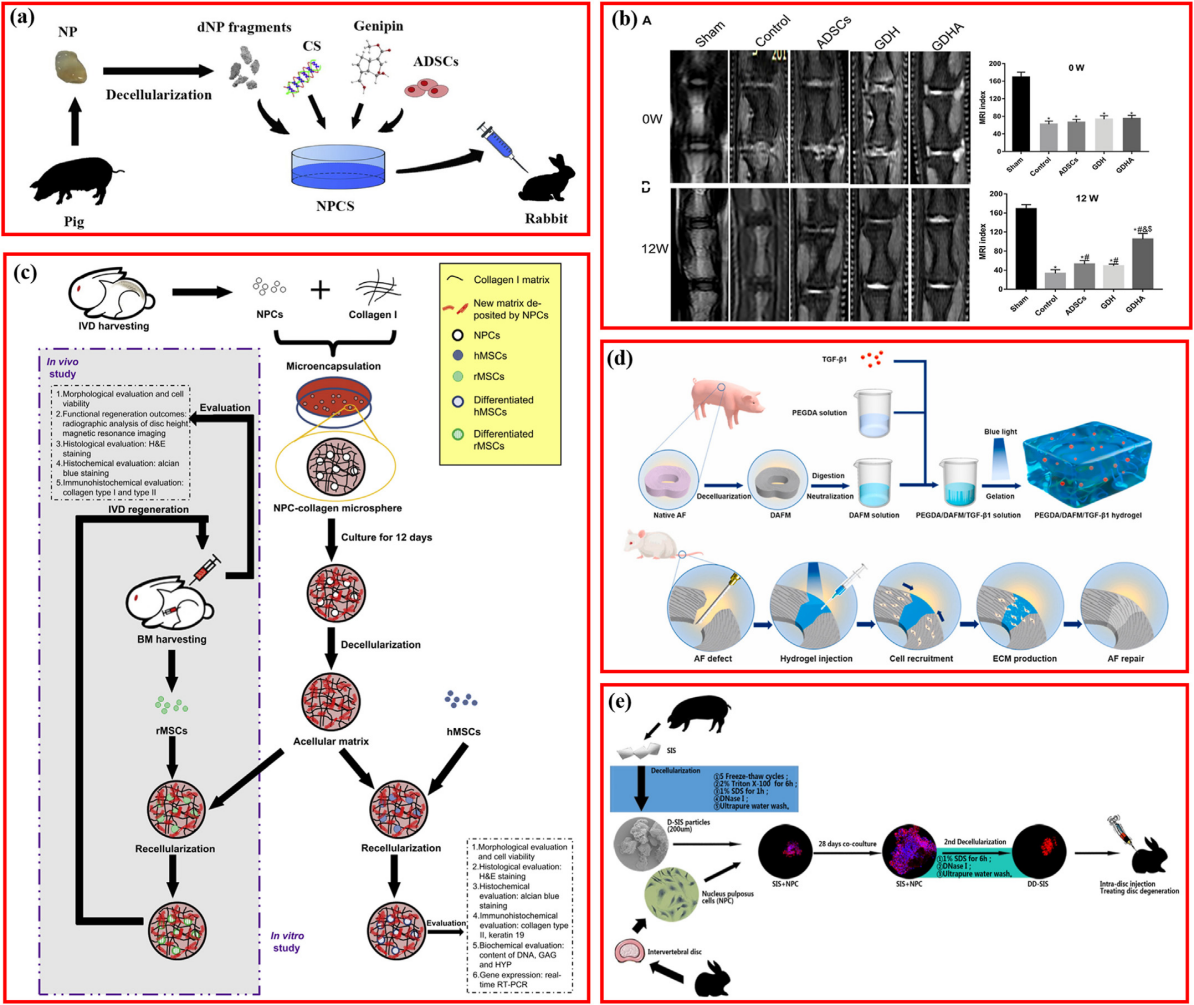
Decellularized matrix reactivated by bioactive factor or cells. (a) Schematic illustration of the fabrication of injectable decellularized nucleus pulposus-based cell delivery system. Adapted with permission from Ref. [15], copyright 2018 Elsevier BV. (b) Typical MRI and MRI index change after injection of decellularized matrix. Adapted with permission from Ref. [96], copyright 2021 Frontiers Media S.A. (c) Effects of nucleus pulposus cell-derived acellular matrix on the differentiation of mesenchymal stem cells. Adapted with permission from Ref. [35], copyright 2013 Elsevier BV. (d) Schematic illustrating fabrication of PEGDA/DAFM/TGF-β1 hydrogels for AF repair by injecting hydrogels. Adapted with permission from Ref. [99], copyright 2022 KeAi Communications Co. (e) Schematic illustrating the fabrication of injectable nucleus pulposus cell-modifed decellularized scaffold. Adapted with permission from Ref. [46], copyright 2017 Impact Journals LLC.

Based on the principle that native is ideal, researchers attempted to restore the microstructure and microenvironment using DCM plus NP or AF cells. Zhou et al. developed a decellularized NP-based cell delivery system (NPCS) containing decellularized NP and ADSCs (Fig. 6a) [15]. The NPCS exhibited similar mechanical properties to the fresh NP tissue and were capable of inducing NP-like differentiation i*n vitro*. Moreover, this cell-delivery system significantly improved the disc height and MRI indexes in vivo, restoring the IDD structure partially. Equally, another 0.02% genipin cross-linked decellularized NP hydrogel-loaded ADSCs (GDHA) demonstrated potential as a therapeutic approach for retarding IDD (Fig. 6b) [96]. To expand this idea, NPCs were cultured in collagen microspheres to produce NPC-derived matrices, and the NPC-derived matrices were repopulated with MSCs for rabbit IDD model repair (Fig. 6c) [35]. This experiment indicated that the NPC-derived matrix was favorable for MSC matrix formation and promoted the expression of NPC markers genes.

Furthermore, the MSC-decorated NPC-derived matrix could repair the IDD model more effectively. However, it is important to note that cell-based treatments may be restricted by undesirable differentiation triggers, immunological rejection, and tumorigenicity [[97], [98], [99]]. Nevertheless, the direct delivery of cells at target locations, cell recruitment, or cellular function enhancement in the site using bioactive factors for tissue repair poses a favorable therapeutic strategy. Among bioactive factors, growth factors were the leading molecules for IVD tissue engineering [58,100,101], of which transforming growth factor-beta 1 and 3 (TGF-β1, TGF-β3) and basic fibroblast growth factor (bFGF) have been integrated into DCM scaffolds to improve IDD repair [58,60,99]. TGF-β1 reportedly downregulates the oxidative stress-induced autophagy and apoptosis of AF cells by regulating the ERK cascade pathway, and its incorporation into scaffolds could initiate the AF-cell functional phenotype [102,103]. TGF-β1 was incorporated into a polyethylene glycol diacrylate/decellularized AF matrix (PEGDA/DAFM) mixture to create a photocrosslinkable, an injectable hydrogel for AF repair [99]. Results indicated that the addition of PEGDA contributed to significantly better mechanical properties of the final PEGDA/DAFM/TGF-β1 hydrogels, and the released TGF-β1 facilitated the migration of AF cells, preventing NP atrophy, retaining disc height, and restoring the disc biomechanics (Fig. 6d). With the same background and aim, TGF-β3 and bFGF were inserted into the decellularized NP matrix/chitosan (DNPM/chitosan) and decellularized AF matrix/chitosan (DAFM/chitosan) hydrogels respectively, revealing that the ornamental TGF-β3 and bFGF induced the secretion of COL I and COL II from NP stem cells and AF stem cells (AFSC) [58,60]. Although the incorporation of single bioactive factors addressed the side effects of cell-based therapy, the resultant curative effect was correspondingly limited because IVD tissues are regulated by several proteins or molecules synergistically deposited in their biological environments. Therefore, another novel DCM-based scaffold was designed. NPCs or stem cells were seeded on the DCM first, and then the composite scaffolds were decellularized again after the secretion and deposition of multiple molecules. In this design, NPCs were first cultured into the DCM derived from porcine SIS. After culturing for 28 days, the composite DCM-cell system was decellularized again to prepare the second decellularized SIS (Fig. 6e), thus, preventing the progression of the rabbit IDD effectively [46]. Hence, the reactivation of DCM with cells or bioactive factors is gaining increasing attention, and it provides more insight into IDD repair. It is noteworthy that IVD progenitor cells, referred to as the native progenitor-like cells and some other proliferative cellular population isolated from the IVD, may provide a new alternative for IDD repair [104]. Furthermore, as an emerging next-generation therapeutic strategy, although exosomes (Exos), also called extracellular vesicles (EVs), have been broadly applied to treat IDD, no study has investigated the Exos-supplemented DCM materials for IDD repair [105].

Acellular matrices are mainly used in two forms, plain DCM and processed DCM, which have respective advantages and disadvantages. The advantages and disadvantages of different DCM for IDD repair are summarized in Table 6. As stated above, plain DCM scaffolds, which inherited the characteristic architecture and hydrogels enjoy the merits of injectability and fluidity in filling irregular defects, and injectable hydrogel must sacrifice the original 3D structure of the disc. Nevertheless, we must be aware of the complexity of the IVD microenvironment and that a single factor may be insufficient to repair IDD. Some scholars proposed that more interest should be paid to the injectability and reservation of the soluble biofactors than the DCM structure [14]. Injectable biomaterials have aroused interest due to their ease of implantation, excellent malleability, and minor damage [106,107]. Theoretically, ideal decellularized hydrogels for IVD repair should possess the following advantages: 1) mimic native IVD tissues in composition and structure, 2) serve as well-developed cellular transmitters with fine biocompatibility, 3) induce the directional differentiation of stem cells, 4) allow application through injection without huge invasion, and 5) restore the hydrostatic compressive resistance of the disc [3,21]. However, it is important to note that leakage of DCM hydrogels would lead to treatment failure [108]. Although decellularization did not change the elastic modulus of AF significantly (from 14.71 ​± ​1.19 to 34.94 ​± ​3.53 ​MPa), the fabrication of an injectable DCM may decrease the modulus considerably [20,92]. Hydrogel-phase DCM exhibited another advantage by reverting desirable mechanics from altering the cross-linking parameters to meet specific requirements, including the cross-linking temperature, time, application of supporting light, and concentration of crosslinkers. For instance, the widely utilized crosslinker, genipin, was validated to show the capability of improving the mechanical properties of multiple biomaterials with a cytotoxic concentration of 0.04% [20,109]. As an upgrading pattern, injecting stem cells or IVD cells were repopulated in the DCM to enhance bioactivity. They are both particularly significant, with acceptable stimulatory effects on directional differentiation. Thus, loading and delivering cells to the target location with DCM has addressed the avascular drawback of the IVD in recruiting cells. Furthermore, reseeding stem cells onto DCM fully utilizes the differentiation potential of DCM and the ability to secrete various factors required for IVD regeneration. DCM supplementation with bioactive molecules to improve IDD repair has been demonstrated to be feasible because this approach avoids the biological uncertainty of cells. Injecting stem cells or IVD cells for IDD regeneration has been extensively studied over the past decade [110,111], even progressing into clinical trials [9].

**Table 6:** Comparison between different types of DCM for IDD repairment.

Advantages and disadvantages					
		Plain DCM	Hydrogel-form DCM	Reactivated DCM	
Advantages		Possessing the initial microstructure and excellent biocompatibility without integrating additional agent.	Possessing injectability and ability to realize free shaping, load cells and factors.	Possessing ideal bioactivity and loading cells directly for excellent integration.	
Disadvantages		Lacking sufficient bioactivity, biodegradability and ability to be shaped relatively.	Lacking structure biomimicking the natural IVDs.	Being difficult to fabricate and raising consideration for biocompatibility due to additional agents.	
**Biocompatibility**					
		Plain DCM	Hydrogel-form DCM	Reactivated DCM	
In vitro		Biocompatible for NPC [1,46,72]; AF cells [1,40,54]; MSCs [38,43,[71], [72], [73], [74],^81^,^89^]; hADSCs [37]; human-derived IDD cells [38,43,44]; fibroblasts [56,115]	Biocompatible for hBMSCs [6,20]; NPC [14], dermal fibroblasts [21]; hADSCs [92]; unbiocompatible for DRG neurons [11],	Biocompatible for hADSCs [15,96]; hMSC [35]; rabbit AFSCs [58,59,116]; AF cells [99]	
In vivo		Biocompatible for rabbit [38,71]; rat [46,73,111]	Biocompatible for rat [92]	Biocompatible for rabbit [35], rat [116]	
**Degradability**					
		Plain DCM	Hydrogel-form DCM	Reactivated DCM	
In vitro		NA	NA	68% collagen and 43% sGAG remained after 30 days [15]	
In vivo		NA	NA	Degraded obviously as time prolonging [116]	
**Mechanical property**					
		Plain DCM	Hydrogel-form DCM	Reactivated DCM	Referred data
Elastic/Storage modulus	AF	1.15–34.94 ​MPa [38,54,56,73]	0.1–3.29 ​MPa [6,14,20]	1.51 ​± ​0.14 ​MPa [59]	18.98–62.58 ​MPa [38,54,56]
	NP	5.85–10.13 ​kPa [74]	150.10 ​± ​3.65 ​Pa [6]	13.71 ​± ​0.53 ​kPa [15]	14.19–42.18 ​kPa [15,74]
Loss modulus	NP	1.68–4.80 ​kPa [74]	NA	6.41 ​± ​0.15 ​kPa [15]	2.12–6.56 ​kPa [15,74]
Toe-region modulus	NP	1.4 ​± ​1.2 ​kPa [37]	NA	NA	1.1 ​± ​0.3 ​kPa [37]
	Total IVD	0.134 ​± ​0.048 ​MPa [36]	NA	NA	0.828 ​± ​0.464 ​MPa [36]
Linear-region modulus	Total IVD	0.640 ​± ​0.360 ​MPa [36]	NA	NA	10.132 ​± ​3.76 ​MPa [36]
	AF	62.0 ​± ​13.6 ​MPa [40]	NA	NA	NA
	NP	11.0 ​± ​4.9 ​kPa [37]	NA	NA	15.9 ​± ​4.0 ​kPa [37]
Equilibrium modulus	NP	1.0 ​± ​0.1 ​kPa [37]	NA	NA	1.5 ​± ​0.6 ​kPa [37]
Relaxation ratio	NP	29.2–83.56% [37,74]	NA	NA	38.4–77.20% [37,74]
Compressive moduli	Total IVD	18.41 ​± ​8.38 ​kPa [38]	NA	NA	20.00–317.2 ​kPa [38,99]
Relaxation time	AF	NA	1.5 ​± ​0.9 ​min [6]	NA	NA
	NP	NA	4 ​± ​0.4 ​min [6]	NA	NA
Rheological properties					
		Plain DCM	Hydro-form DCM	Reactivated DCM	Referred data
Dynamic shear modulus	AF	NA	400–530 ​Pa [11]		7.4–19.8 ​kPa [11]
	NP	2.56–4.44 ​kPa [74]	NA	NA	11.00–18.73 ​kPa [74]
tan ​δ	AF	NA	0.4–0.7 [11]	NA	0.424–0.577 [11]

NA: Not available; NP: nucleus pulposus; AF: annular fibers; NPC: nucleus pulposus cell; hADSCs: human adipose-derived stem cells; bFGF: basic fibroblast growth factor; TGF-β1: transforming growth factor β1; NPCS: NP-based cell delivery system.

For implanted biomaterials, biocompatibility was regarded as the premise for clinical application. According to existing reports, no notable in vitro or in vivo biotoxicity was reported in DCM and derived scaffolds (Table 6). Both plain and processed DCM demonstrated to be biocompatible in various cells.

Mechanical properties are another key index of DCM scaffolds. The elastic modulus of the plain, hydrogel-form, and reactivated AF DCM scaffolds were reported to be 1.15–34.94 ​MPa [38,54,56,73], 0.1–3.29 ​MPa [6,14,20], and 1.51 ​± ​0.14 ​MPa [59], respectively, and only the plain AF DCM was consistent with the range of natural AF tissue (18.98–62.58 ​MPa) [38,54,56]), as listed in Table 6. Regarding the other mechanical properties, including the loss modulus, toe-region modulus, linear-region modulus, relaxation ratio, and compressive modulus, data were only reported for the plain DCM, which were similar to native tissues. However, the rheological properties of decellularized AF DCM were only evaluated in the hydrogel-form DCM, and the dynamic shear modulus and tan ​δ were 400–530 ​Pa and 0.4–0.7, respectively [11]. The plain decellularized NP DCM demonstrated a dynamic modulus of 2.56–4.44 ​kPa [74]. Consistent with natural tissues, the mechanical modulus of AF is lower than that of NP. Although the mechanical performance of the hydrogel-form DCM could be modified though regulating the processing parameters, both the hydrogel-form AF and NP DCM possessed weaker mechanical values than did the plain DCM. All reported mechanical parameters are listed in Table 6; future studies should attempt to develop DCM scaffolds approaching these data to better repair IDD.

**Fig. 7:**
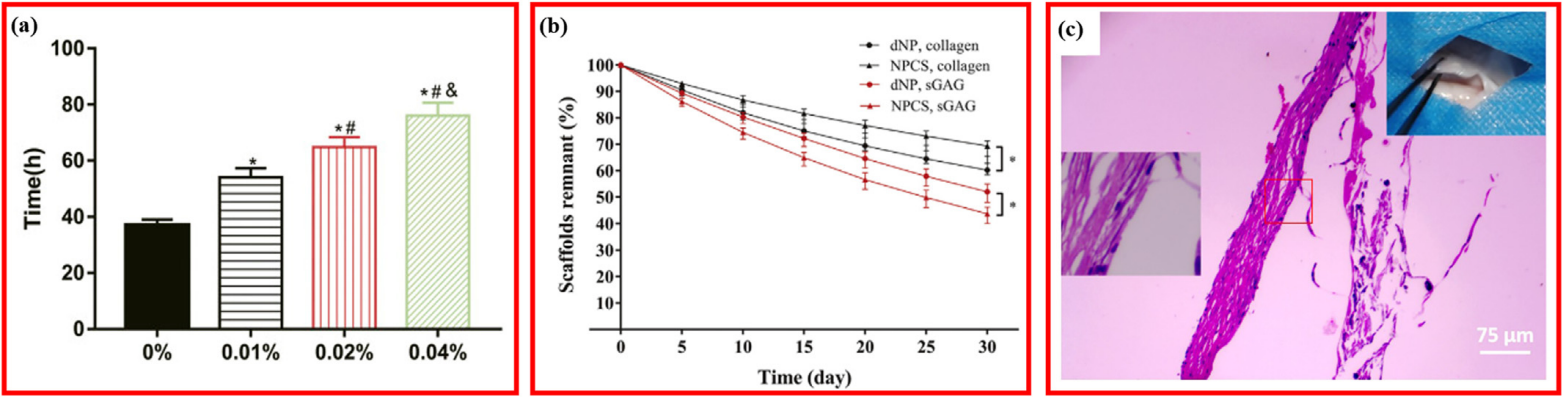
Degradability in decellularized materials for repairing IVD. (a) *In-vitro* tability of GDH cross-linked with different-concentration genipin. Adapted with permission from Ref. [96], copyright 2021 Frontiers Media S.A. (b) sGAG and collagen remnants of decellularized nucleus pulposus cross-linked with 0.02% genipin. Adapted with permission from Ref. [15], copyright 2018 Elsevier BV. (c) Hematoxylin and eosin staining of the DAFM/PECUU2-blended fibrous scaffolds after implantation for 12 weeks. Adapted with permission from Ref. [112], copyright 2022 John Wiley and Sons Inc.

The degradability or biostability of the DCM-derived scaffolds were usually measured after treatment using an enzymatic digestion solution. As expected, no studies reported the degradability of the plain DCM. Both the in vitro and in vivo degradabilities of the reactivated composite DCM scaffolds were investigated. As shown in Fig. 7a, the stability of GDHA increased significantly as the cross-linking extent increased [96]. In NPCS crosslinked with 0.02% genipin, after degradation for 30 days, nearly 68% of the collagen and 43% of the sGAG remained, and the ratio of sGAG-to-hydroxyproline was notably higher than that of pure dNP (Fig. 7b) [15]. When the GDH was implanted subcutaneously, the inflammatory cell infiltration was reduced and the DAFM/PECUU-blended fibrous scaffolds thinned significantly at 12 weeks, indicating the degradability of the scaffolds (Fig. 7c) [112]. Thus, reactivated composite DCM scaffolds showed great advantages in the biodegradability compared with plain or hydrogel-form DCM.

In addition to the properties discussed above, another characteristic, the osmotic pressure of DCM, deserves attention. The normal physiologic function of the NP relies heavily upon the osmotic pressure, which provides the NP with the ability to withstand the extensive axial compressive load, serving as a buffer, and prevents the ingrowth of blood vessels and nerves into the IVD [14,37]. During IDD, the osmotic pressure increases [113]. The osmotic pressure affects the time-dependent recovery of the IVD [114] and plays an important role in the bioactivity of NP mesenchymal stem cells (NPMSCs), which was mediated via the P16^INK4A^/Rb pathway [113]. Interestingly, the osmotic pressure was derived from the swelling of the sGAG that was abundant in the NP [11,115]. The osmotic activity was only dependent on the water outside the fiber [116]; thus, the water content is a critical index for the osmotic pressure. The water content of fresh NP tissue was 76.58 ​± ​1.47% to 88.7 ​± ​0.7%; the water content of decellularized NP could increase by 5–17.98% and could be as high as 98.5% [37,71,74]. It was suggested that the increasing water content was attributable to the disruption of peptide bonds, which resulted in more exposed carboxylic and amino groups connecting with free water using hydrogen bonds [71,117]. Zhou et al. reported that the NP water content decreased during decellularization, and the addition of genipin could increase the content to a level similar to that of fresh tissues [15]. Decellularized NP scaffolds demonstrated a swelling ratio of 272.16 ​± ​33.30% [89] to 300% [14].

## 4 Mechanism involving the biological activity of DCM in repairing IDD

**Fig. 8:**
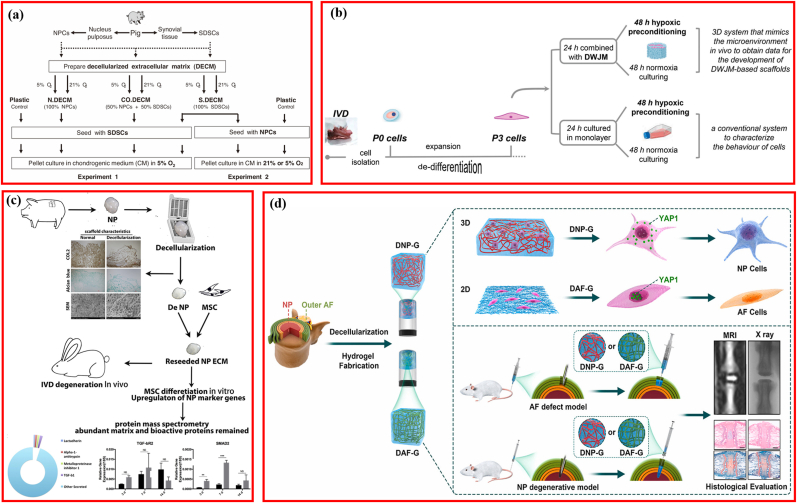
Signaling pathway involving the biological activity of decellularized matrix on interverbal disc degeneration. (a) Modulating the microenvironment of tissue-specific cell-based decellularized matrix with low oxygen to facilitate NP tissue regeneration. Adapted with permission from Ref. [121], copyright 2012 Lippincott Williams and Wilkins Ltd. (b) Combination of decellularized matrix and hypoxic priming improving the phenotype of degenerate intervertebral disc cells. Adapted with permission from Ref. [44], copyright 2022 Elsevier Inc. (c) NP-like cells differentiation of stem cell via the TGF-β signaling pathway. Adapted with permission from Ref. [71], copyright 2019 Elsevier. (d) Schematic illustrating decellularized matrix fabrication and in vitro and in vivo applications. Adapted with permission from Ref. [6], copyright 2021 KeAi Communications Co.

Satisfactory IDD repair using DCM was accessed; however, the underlying signaling pathway mediating the therapeutic efficacy remained rarely elucidated. Clarification of the molecular cascade contributed to further fabrication, design, development, and modification of DCM-based biomaterials for IDD repair. Several studies have attempted to illustrate the signaling pathways involved in IVD regeneration (Fig. 8), which are listed in Table 7.

**Table 7:** Mechanisms involving the biological activity of DCM in repairing IVD.

Author	Source	Preparing condition	Style of Matrix	Seeding Cell	Animal model	Culturing condition	Key molecule	Findings
Pei et al. [121]	Porcine SDSC	Normoxia/hypoxia	Pellet	SDSC/NPC	NA	Normoxia/hypoxia	NA	Low oxygen in a pellet culture system enhanced NPC viability and redifferentiation.
	Porcine SDSC ​+ ​NPC	Normoxia/hypoxia	Pellet	SDSC		Hypoxia	NA	
	Porcine NPC	Normoxia/hypoxia	Pellet	SDSC		Hypoxia	NA	
Xu et al. [71]	Porcine NP	NA	Hydrogel	MSCs	Rabbit/IVD degeneration	NA	TGF-β 1	Decellularized NP-ECM promoted MSCs to differentiate into NP-like cells and rescued the degenerated IVD through activating the TGF-β signaling pathway
Peng et al. [6]	Bovine NP	NA	Hydrogel	hBMSCs	Rabbit/NP degeneration	3D	Integrin	Decellularized NP and AF matrix could induce hBMSCs differentiation through the ​integrin-mediated RhoA/LATS/YAP1 signaling pathway
	Bovine AF	NA	Hydrogel	hBMSCs	Rabbit/AF defect	2D		
Penolazzi et al. [44]	Human DWJM	NA	Scaffold	Degenerate IVD cells	NA	Hypoxic priming	HIF-1α	When exposed to hypoxic priming and DWJM, Degenerate IVD cells could re-adapt to hypoxia with HIF-1α recruited at the promoter of SOX9 and FOXO3.
Liu et al. [59]	Rabbit AF	NA	Nanofibrous scaffolds	AFSCs	NA	NA	NA	DAFM/PECUU electrospun scaffolds were potential candidate for AF tissue engineering applications.
Liu et al. [112]	Rabbit AF	NA/Varying elastic moduli	Nanofibrous scaffolds	AFSCs	Rat/Annulus fibrosus defects	NA	COL Ⅰ	DAFM/PECUU-blended scaffolds could effectively repair AF defect and closely mimic the biological and mechanical properties of native tissue.

NP: nucleus pulposus; AF: annular fibers; NPC: nucleus pulposus cell; IVD: intervertebral disc; SDSC: synovium-derived stem cells; AFSC: AF stem cells.

The role of oxygen in IVD cells remains controversial, with some evidence indicating that reduced oxygen supply is associated with IDD, whereas some scholars suggest that hypoxia is essential for the maintenance of the physiological function of the IVD [[118], [119], [120]]. In vitro microenvironments (oxygen concentration and cell source) in DCM preparation influenced cellular proliferation and differentiation to NPCs; DCM fabricated under hypoxia favored viability and redifferentiation of NPCs [121]. To further explore the effect of culturing oxygen concentration on the expansion and redifferentiation of NPCs, the cells were seeded on DCM derived from synovium-derived stem cells and cultured in either hypoxia (5% O_2_) or normoxia (21% O_2_). Enhanced NPC redifferentiation was observed in hypoxic circumstances (Fig. 8a) [121]. Moreover, degenerate IVD cells seeded in DWJM scaffolds readapted and regained the discogenic phenotypes in response to hypoxic priming, with HIF-1α specifically recruited to the crucial genes in IVD homeostasis and repair (SOX9 and FOXO3a) [44]. These results indicated that hypoxia is a critical factor for IDD repair (Fig. 8b). TGF-β1, which is crucial for NPC differentiation, was screened in the decellularized NP with protein mass spectrometry, and the upregulated expression levels of the downstream cascading genes (SMAD2 and SMAD3) were validated using real-time reverse-transcription polymerase chain reaction detection, suggesting that the TGF-β signaling pathway is responsible for the facilitation effect of decellularized NP on MSC differentiation into NP-like cells (Fig. 8c) [71]. Intriguingly, it was reported that decellularized AF hydrogel (DAF-G) and NP hydrogel (DNP-G) directed hBMSCs to differentiate into hBMSCs AF- and NP-like cells, respectively, via the RhoA/LATS/YAP1 molecular pathway mediated by integrin. However, YAP1 played an opposite regulatory role on tissue-specific differentiation of hBMSCs initiated by DAF-G and DNP-G, which inhibited differentiation into NP-like cells and promoted differentiation into AF-like cells (Fig. 8d) [6]. The spatial structure and biomechanical properties of DCM are important for IVD regeneration [6,122,123].

Decellularized AF matrix/poly (ether carbonate urethane) urea (DAFM/PECUU) electrospun scaffolds were prepared via coaxial electrospinning, which promoted the secretion of COL I, COL II, and aggrecan from AFSC [59]. Further studies have demonstrated that *COL I* expression increased as the elasticity of the DAFM/PECUU scaffold increased, whereas the opposite was observed in the case of *COL II* and *aggrecan*, indicating that the elasticity of the scaffold is a vital regulator of AFSC differentiation [112].

Although the DCM reportedly induces AF cell-like differentiation, no acknowledged index was addressed. Moreover, although *COL1A1* was the most widely used gene marker of AF cells, *COL5A1* was also suggested as a potential marker of AF cells [20,124]. Several signaling pathways have been identified to mediate the therapeutic efficiency of DCM on IDD and its differentiation-inducing effect on IVD cells. DCM promotes stem cell differentiation via the integrin-mediated RhoA/LATS/YAP1 pathway [6]. YAP1 is a transcription coactivator regulating cellular proliferation and differentiation and tissue homeostasis [125], the aggregation of which is associated with AF-like differentiation in the nucleus [126]. Another study indicated that the differentiation instructions from the DCM to MSCs was attributable to the TGF-β signaling pathway [71], and researchers incorporated TGF-β1 into composite hydrogels to improve the regenerative power [99]. As an intersection, TGF-β1 mediates the activation of YAP1 through the PI3K/Akt pathway [6,127], and subsets of the YAP-targeting genes greatly depend on endogenous TGF-β1 [128]. Therefore, TGF-β signals were involved in the MSC differentiation into IVD cells by upregulating *COL II* and *AGN* [38]; thus, the “YAP/TGF-β1” cascade may account for the biological performance of DCM during IDD repair. The higher elastic modulus was associated with differentiation into an AF-specific phenotype [20], while the lower elastic modulus favored NP-like differentiation [15]. Therefore, the elastic modulus could be regulated to facilitate IVD cell differentiation. However, balancing the stiffness of the scaffold presents a major challenge. We speculate that scaffolds with a gradient-distributed elastic modulus are ideal, perhaps because they exhibit a high modulus in the surroundings and a low modulus in the core, which could induce the spatially specific differentiations of IVD cells. Interestingly, decreasing the elastic modulus was associated with the downregulation of total YAP [129,130]. Synthetically, in a closed loop, the elastic modulus and YAP pathway are connected in series, accounting for the bioactivity of DCM in IDD repair.

## 5 Animal models

Animal models are crucial for understanding the IDD progression process and evaluating potential therapies [131]. Moreover, suitable animal models possess higher authority and better credibility. Most studies have employed rabbits to demonstrate the DCM matrix for IDD repair [6,15,35,38,46,72], and a few have used rats [96,99,112] or beagles [66]. Regarding the segments of the IDD model, the lumbar segments are usually chosen in rabbits and beagles [35,38,66], while the coccygeal segments are chosen in rats [96,99,112]. Regarding the approach to create the IDD model, structural puncturing with a fine needle was used in most studies [6,15,20,35,38,46,71,96,99,112]. In detail, an NP degenerative model could be prepared as follows: after anesthesia with 2% isoflurane [15], pentobarbital sodium [20], or 8% chloral hydrate [71], the coccygeal vertebrae (Co4/5, Co5/6, Co7-9) or lumbar vertebrae (L2-L3, L3-L4, L4-L5) of animals (rat [6,96] or rabbit [15,35,71]) were located via manual palpation and confirmed using X-ray imaging. The spine was exposed via an anterolateral retroperitoneal approach, and the IVD was punctured using a sterile needle (16-21G) at a depth of 5 ​mm, with the needles rotated 360° and held for 30 ​s. After closing the wound in layers, animals could freely mobilize, and the prepared degenerative model was regarded as completed immediately [6], 2 weeks later [96], or 4 weeks later [15,35,71], which could be validated by the signal change of MRI within the disc and collapse of disc space. In terms of AF degenerative models, caudal vertebrae (Co4/5, Co5/6, Co7/8, Co8/9) of male rats were aspirated by 18-20G sterile needles at a depth near the border of the inner AF, with NP intact [6,20,99,112]. Regarding the total IVD degeneration model, in addition to puncturing [38,46], surgical removal was also reported [66]. Briefly, after anesthesia, the lower lumbar segments of beagles were exposed. The posterior margin and intervertebral foramen of the target IVD were determined, osteotomy was carried out, and the disc was removed [66]. The animal models of IDD have been summarized by Daly C. et al. [131]. However, some drawbacks existed in these animal models. First, all the animal models were prepared in the non-orthostatic position, which could not simulate human IDD effectively. Furthermore, the coccygeal segments, which bear very little weight, were strikingly different from those in an actual human situation. Although the lumbar segment model could simulate human IDD to some extent, the direction of the mechanical stimulation was nearly perpendicular to the human body. Second, the animals used in these models were small, with the largest being beagles, and there was a lack of large animals, especially primates. Third, some studies implanted the DCM scaffold immediately after the preparation of the model, and this was inconsistent with the pathomechanism of human IDD, which develops and worsens gradually. Fourth, all the animal models had some structural defects, although the main aim was to assess the ability of DCM scaffolds to regenerate IVD.

Adult human IVD is characterized by the absence of a vascular structure, an increased size, reduced notochordal cells, and an exposure to biomechanical stress caused by bipedalism [131]. Owing to the complexity of human IDD, none of the developed animal models could perfectly simulate the overall process pathophysiologically and mechanically. To address this limitation, animal models should be modified to express the aforementioned characteristics. First, chemically induced animal models initiated by various drugs should be used [132]. Second, larger animals, including sheep [133] and goats [134], should be used, as well as primates, such as baboons [135]. The preparation of animal models should mimic the pathology of human IDD as much as possible.

## 6 Perspective and further direction

Significant advancements have been achieved in the use of DCM for IDD repair. Decellularization procedures were modified creatively, and attractive new methods were introduced. However, no comparison was reported between the optimized/developed and primary methods. The main aim of decellularization is to achieve a balance between the complete removal of cells and the ample preservation of the ECM components and architecture. From our perspective, decellularized total IVD may be more favorable for IVD regeneration. Furthermore, bioprinting using DCM-derived bio-ink deserves attention because bioprinting possesses the advantages of an individualized design [[61], [62], [63], [64]]. In further studies, DCM for IDD repair should focus on the following points: decorating the DCM via combing stem cells, NPC, and AFC; modifying the decellularization approaches to optimize the mechanics of DCM, especially the elastic modulus; and designing applicable animal models mimicking the pathological process of human IDD to evaluate the DCM.

## 7 Conclusion

The fabrication procedures of DCM for IDD repair have matured considerably, and the prepared materials exhibit excellent in vitro biocompatibility and potent in vivo therapeutic efficiency for IDD, with plain and processed scaffolds being the most common. Furthermore, considerable developments have been achieved in DCM reactivation and the elucidation of potential mechanisms. However, further studies are needed to design animal models simulating the human body and to evaluate the curative effect of DCM in clinical trials.

## Author contributions

JA and ZY conceived the original ideas of this manuscript and reviewed the finished manuscript and executed supervision throughout the process. HQ and LH prepared the manuscript, tables, and figures. ZW made critical revision for the manuscript. All authors have read and approved the manuscript.

## Declaration of competing interest

The authors declare that they have no known competing financial interests or personal relationships that could have appeared to influence the work reported in this paper.

## Data Availability

No data was used for the research described in the article.
